# Usage Patterns of Web-Based Stroke Calculators in Clinical Decision Support: Retrospective Analysis

**DOI:** 10.2196/28266

**Published:** 2021-08-02

**Authors:** Benjamin Kummer, Lubaina Shakir, Rachel Kwon, Joseph Habboushe, Nathalie Jetté

**Affiliations:** 1 Department of Neurology Icahn School of Medicine at Mount Sinai New York, NY United States; 2 Clinical Informatics Mount Sinai Health System New York, NY United States; 3 MD Aware LLC New York, NY United States; 4 Ro New York, NY United States; 5 Department of Emergency Medicine Weill Cornell Medicine New York, NY United States; 6 Department of Population Health Science and Policy Icahn School of Medicine at Mount Sinai New York, NY United States

**Keywords:** medical informatics, clinical informatics, mhealth, digital health, cerebrovascular disease, medical calculators, health information, health information technology, information technology, economic health, clinical health, electronic health records

## Abstract

**Background:**

Clinical scores are frequently used in the diagnosis and management of stroke. While medical calculators are increasingly important support tools for clinical decisions, the uptake and use of common medical calculators for stroke remain poorly characterized.

**Objective:**

We aimed to describe use patterns in frequently used stroke-related medical calculators for clinical decisions from a web-based support system.

**Methods:**

We conducted a retrospective study of calculators from MDCalc, a web-based and mobile app–based medical calculator platform based in the United States. We analyzed metadata tags from MDCalc’s calculator use data to identify all calculators related to stroke. Using relative page views as a measure of calculator use, we determined the 5 most frequently used stroke-related calculators between January 2016 and December 2018. For all 5 calculators, we determined cumulative and quarterly use, mode of access (eg, app or web browser), and both US and international distributions of use. We compared cumulative use in the 2016-2018 period with use from January 2011 to December 2015.

**Results:**

Over the study period, we identified 454 MDCalc calculators, of which 48 (10.6%) were related to stroke. Of these, the 5 most frequently used calculators were the CHA_2_DS_2_-VASc score for atrial fibrillation stroke risk calculator (5.5% of total and 32% of stroke-related page views), the Mean Arterial Pressure calculator (2.4% of total and 14.0% of stroke-related page views), the HAS-BLED score for major bleeding risk (1.9% of total and 11.4% of stroke-related page views), the National Institutes of Health Stroke Scale (NIHSS) score calculator (1.7% of total and 10.1% of stroke-related page views), and the CHADS_2_ score for atrial fibrillation stroke risk calculator (1.4% of total and 8.1% of stroke-related page views). Web browser was the most common mode of access, accounting for 82.7%-91.2% of individual stroke calculator page views. Access originated most frequently from the most populated regions within the United States. Internationally, use originated mostly from English-language countries. The NIHSS score calculator demonstrated the greatest increase in page views (238.1% increase) between the first and last quarters of the study period.

**Conclusions:**

The most frequently used stroke calculators were the CHA_2_DS_2_-VASc, Mean Arterial Pressure, HAS-BLED, NIHSS, and CHADS_2_. These were mainly accessed by web browser, from English-speaking countries, and from highly populated areas. Further studies should investigate barriers to stroke calculator adoption and the effect of calculator use on the application of best practices in cerebrovascular disease.

## Introduction

Since the introduction of the Health Information Technology for Economic and Clinical Health Act in 2009, hospital systems in the United States have seen a five-fold increase in electronic health record (EHR) system adoptions [1,2]. These increases in EHR adoption have been accompanied by an upsurge in the amount of clinical data contained in EHRs. Providers’ increasingly challenging task of managing this growing amount of information may result in cognitive burdening [3]. Moreover, the manner in which many EHRs display large amounts of clinical information may not support optimal cognitive reasoning [4]. Providers that use EHRs may therefore experience a number of unwanted adverse effects, including reductions in situational awareness, increases in mental workload, and reduced cognitive performance [5].

Clinical decision support (CDS) systems endeavor to enhance health care delivery by providing clinician-facing and patient-facing information that can improve decision-making at key steps in the workflow [6]. CDS systems are common in modern EHRs and range from passive banners to modal alerting systems for clinical conditions and adverse drug interactions [7,8]. Given that they are capable of delivering variably complex and tailored clinical content at the point of care [9], CDS systems are also well-suited for reducing cognitive overload. Medical calculators are specialized CDS instruments that incorporate user-entered clinical parameters to compute the discrete output of various types of functions [6,10], including physiological equations, risk stratification scores, and disease-quantifying or disability-quantifying scales. While medical calculators are increasingly prevalent in the growing armamentarium of CDS solutions available to providers, few studies have investigated their use patterns and barriers to adoption [5,10,11].

Stroke is a leading cause of disability and mortality worldwide, imposing a heavy economic and public health burden [12,13]. Several clinical scoring systems that draw on clinical, demographic, and laboratory parameters to predict risk, determine disease severity, or grade disability are widely available for the evaluation and management of stroke [14-28]. While medical calculators lend themselves naturally to such use cases, there is a lack of studies describing the current state of medical calculator use in stroke and cerebrovascular disease. Considering this and the need to better understand the adoption and use of medical calculators, we sought to study the use patterns of frequently used stroke calculators from a widely used web platform.

## Methods

We conducted a retrospective, descriptive study of medical calculators published by MDCalc (MD Aware LLC, New York, NY, USA), a free, web-based and mobile app–based CDS platform that is used by over 65% of US-based physicians monthly and millions of clinicians worldwide [29]. MDCalc’s CDS tools consist of medical score calculator forms for over 200 clinical conditions that allow users to input clinical variables and visualize clinical score outputs, along with an interpretation of the output and an appraisal of the available evidence supporting the use for each score (Multimedia Appendix 1) [6,29].

We used MDCalc’s analytics platform to identify all calculators that were accessed between January 1, 2016 and December 31, 2018. We extracted calculator names; number of cumulative, nonunique page views; mode of access (eg, mobile app or web page); page view ranks; and calculator metadata, including launch dates and structured disease area categories (ie, “tags”). Page view ranks were assigned for each calculator based on total page views over the study period, with the lowest rank corresponding to the highest number of page views. Each calculator’s cumulative page views were expressed relative to total cumulative page views for the entire MDCalc platform over the study period.

We defined calculators related to stroke as any calculator that contained 1 or more stroke-related tag (ie, “ischemic stroke,” “transient ischemic attack,” “intracerebral hemorrhage,” or “subarachnoid hemorrhage”). For the 5 calculators with the highest relative page views over the study period, we determined quarterly page views, page views stratified by mode of access (eg, web page, iOS mobile app, or Android mobile app), country, and US state. For each calculator, we additionally determined page views relative to all stroke-related calculators and calculated the rate of increase in relative page views between the first and last quarter of the study period. To describe the evolution in stroke-related calculator use and rankings in the 5 years prior to the start of the study period, we determined relative page views and ranks for the same 5 calculators between January 1, 2011 and December 31, 2015. We then compared these measurements to those for the 2016-2018 study period. We only included calculators that were published by MDCalc.

## Results

Between January 1, 2016 and December 31, 2018, we identified 454 MDCalc calculators, of which 48 (10.6%) were related to stroke. By cumulative page view, the 5 most highly ranked stroke calculators were the CHA_2_DS_2_-VASc (congestive heart failure, hypertension, 75 years of age and older, diabetes mellitus, previous stroke or transient ischemic attack, vascular disease, 65 to 74 years of age, female) score for atrial fibrillation stroke risk calculator (5.5% of total MDCalc and 32% of stroke-related page views), the Mean Arterial Pressure (MAP) calculator (2.4% of total MDCalc and 14% of stroke-related page views), the HAS-BLED (hypertension, abnormal renal/liver function, stroke, bleeding history or predisposition, labile international normalized ratio, elderly, drugs/alcohol concomitantly) score for major bleeding risk calculator (1.9% of total MDCalc and 11.4% of stroke-related page views), the National Institutes of Health Stroke Scale (NIHSS) score calculator (1.7% of total MDCalc and 10.1% of stroke-related page views), and the CHADS_2_ (congestive heart failure, hypertension, 75 years of age or older, diabetes mellitus, and previous stroke or transient ischemic attack) score for atrial fibrillation stroke risk calculator (1.4% of total MDCalc and 8.1% of stroke-related page views; Table 1). 

**Table 1:** Relative page views and ranks of the 5 most frequently used MDCalc stroke calculators, 2011-2018.

				2011-2015^a^				2016-2018^b^		
Calculator	Description^c^	Launch date	Proportion of all calculator page views, %^d,e^		Proportion of stroke calculator page views, %^d,f^	Rank^g^	Proportion of all calculator page views, %^d,e^		Proportion of stroke calculator page views, %^h^	Rank^g^
CHA_2_DS_2_-VASc^i^	Calculates stroke risk for patients with atrial fibrillation, possibly better than the CHADS_2_^j^ score	April 1, 2011	4.9		38.7	2	5.5		32	2
MAP^k^	Calculates mean arterial pressure	January 1, 2009	1.1		8.6	31	2.4		14	7
HAS-BLED^l^	Estimates risk of major bleeding for patients on anticoagulation to assess risk-benefit in atrial fibrillation care	April 1, 2011	2.2		17.4	12	1.9		11.4	9
NIHSS^m^	Calculates the NIH^n^ Stroke Scale for quantifying stroke severity	January 1, 2009	1.0		7.7	33	1.7		10.1	15
CHADS_2_	Estimates stroke risk in patients with atrial fibrillation	January 1, 2009	2.9		22.6	7	1.4		8.1	22

^a^The 2011-2015 period is from January 1, 2011 to December 31, 2015.

^b^The 2016-2018 period is from January 1, 2016 to December 31, 2018.

^c^Descriptions are as appears on each MDCalc calculator webpage.

^d^All page views exclude Android/iOS MDCalc app page views.

^e^Percentage is relative to page views for all MDCalc calculators available during specified period.

^f^Percentage is relative to page views for 22 stroke-related calculators available during specified period.

^g^Rank is assigned according to cumulative, nonunique MDCalc page views relative to all available MDCalc calculator page views for each specified period; lowest rank corresponds to the highest proportion of page views.

^h^Percentage is relative to page views for 48 stroke-related calculators available during specified period.

^i^CHA_2_DS_2_-VASc: congestive heart failure, hypertension, 75 years of age and older, diabetes mellitus, previous stroke or transient ischemic attack, vascular disease, 65 to 74 years of age, female.

^j^CHADS₂: congestive heart failure, hypertension, 75 years of age or older, diabetes mellitus, and previous stroke or transient ischemic attack.

^k^MAP: mean arterial pressure.

^l^HAS-BLED: hypertension, abnormal renal/liver function, stroke, bleeding history or predisposition, labile international normalized ratio, elderly, drugs/alcohol concomitantly.

^m^NIHSS: National Institutes of Health Stroke Scale.

^n^NIH: National Institutes of Health.

Native English-language countries accounted for the highest proportion of page views for all calculators. Among individual countries, the United States, followed by the United Kingdom, accounted for the highest proportion of page views for all calculators except for the CHADS_2_ score, for which Canada accounted for the second-highest proportion of page views. Within the United States, the states of California, Texas, New York, Pennsylvania, and Florida accounted for the highest proportion of page views for all calculators except the MAP score, for which Washington, California, Oregon, Texas, and New York accounted for the greatest share. Among individual states, the highest proportion of page views originated from California for the CHA_2_DS_2_-VASc, NIHSS, and CHADS_2_ scores, whereas the highest number of page views originated from New York for the HAS-BLED score and Washington for the MAP score. Use patterns for the NIHSS calculator are shown in Table 2, which shows similar use patterns as for the CHA_2_DS_2_-VASc, HAS-BLED, and CHADS_2_ score calculators. The MAP calculator use pattern is represented separately in Table 3.

**Table 2:** Growth in relative page views of the National Institutes of Health Stroke Scale score calculator by quarter and year.

Quarter (year)	Proportion of total page views, %
Q1 (2016)	4.2
Q2 (2016)	4.3
Q3 (2016)	6.6
Q4 (2016)	4.7
Q1 (2017)	6.7
Q2 (2017)	6.5
Q3 (2017)	7
Q4 (2017)	8.8
Q1 (2018)	10.8
Q2 (2018)	12.2
Q3 (2018)	13.9
Q4 (2018)	14.2

**Table 3:** Growth in relative page views of the Mean Arterial Pressure score calculator by quarter and year.

Quarter (year)	Proportion of total page views, %
Q1 (2016)	5.1
Q2 (2016)	5.4
Q3 (2016)	8.5
Q4 (2016)	7
Q1 (2017)	7.2
Q2 (2017)	7.5
Q3 (2017)	8
Q4 (2017)	8.5
Q1 (2018)	9.9
Q2 (2018)	10
Q3 (2018)	10.6
Q4 (2018)	12

All 5 calculators were predominantly accessed by web browser rather than by mobile apps. The proportion of access attributable to web browsers varied depending on the specific calculator. However, web browser access accounted for 82.7%-91.2% of frequently used stroke calculator page views, with the NIHSS and MAP calculators respectively representing the minimum and maximum in the range. The NIHSS calculator had the highest proportion of Android app page views (10.7%). Two calculators, the NIHSS and CHA2DS2-VASc, generated the highest and equal proportion of iOS app pageviews (6.6%) (data not shown). The NIHSS score calculator demonstrated the greatest increase in page views (238.1% increase) between the first and last quarters of the study period (Table 2).

All 5 calculators were released by MDCalc between January 2009 and April 2011. In chronological order, the CHADS_2_ score and MAP calculators were released the earliest (January 1, 2009), followed by the NIHSS calculator (January 1, 2011) and the HAS-BLED and CHA_2_DS_2_-VASc score calculators (April 1, 2011). Over the study period, the CHA_2_DS_2_-VASc score calculator was ranked 2nd; MAP, 7th; HAS-BLED, 9th; NIHSS, 15th^;^ and CHADS_2_, 22nd. By contrast, between January 2011 and December 2016, the corresponding ranks for these calculators were 2nd, 31st, 12th, 33rd, and 7th, respectively (Table 1).

## Discussion

### Principal Findings

In this study, we found that the most frequently accessed calculators relating to stroke comprised 1 of 3 types: risk prediction tools for complications that were conditional on the presence of a specific disease state (eg, CHADS_2_, HAS-BLED, and CHA_2_DS_2_-VASc scores), scales to quantify severity in ischemic stroke (eg, NIHSS), and calculators for computing physiologic parameters (eg, MAP). These calculators were among the most frequently used calculators on the MDCalc platform, as demonstrated by the CHA2DS2-VASc score calculator that ranked second by relative page views in both the 2011-2015 and 2016-2018 periods and by the increases in ranks observed in all stroke calculators during the 2016-2018 period. The majority of the calculators were accessed from the most highly populated US states [30] with the greatest number of licensed physicians [31]. While a number of page views did originate from outside the United States, most of these, nonetheless, originated from English-language countries.

### Characteristics of Highly Used Stroke Calculators

#### English-Language Dominance and Association With High-Prevalence Conditions

Many drivers of stroke calculator use that we uncovered in our analysis may also be generalizable features of highly used calculators outside the field of stroke. One primary such driver may be the predominance of the English language, which is best exemplified by our findings that the highest rates of geographical calculator use originated in English-language countries. However, potential additional factors contributing to the predominance of English in calculator use include the widespread use of English in scientific and clinical communities worldwide [32], the fact that MDCalc has an English-only website [29] and was founded by 2 US emergency medicine physicians, and the platform’s primarily word-of-mouth advertising strategy in English-language countries. A second potentially generalizable feature of highly used calculators is high disease prevalence. Our findings demonstrate that 3 of the 5 (60%) most highly used calculators related to atrial fibrillation, which is both highly prevalent in elderly patients [33] as well as patients with ischemic stroke [34]. As suggested by our findings, calculators addressing highly prevalent diseases may be likely to generate higher use.

#### Inclusion in Professional Society Guidelines

A third potentially generalizable feature of calculators is their inclusion of corresponding scores in professional society guidelines, as shown in our study by both CHA_2_DS_2_-VASc and HAS-BLED. The former score was incorporated into US and international professional society guidelines for the management of atrial fibrillation, including the European Society of Cardiovascular in 2012 and 2016 [35,36], the American Heart Association in 2014 [37], the National Institute for Health and Care Excellence United Kingdom guidelines in 2014 [38], and the Asia Pacific Heart Rhythm Society guidelines in 2017 [39]. Similarly, the HAS-BLED score was incorporated in European Society of Cardiovascular in 2012 and 2016 [35,36], the Canadian Cardiovascular Society in 2014 and 2018 [40,41], and the National Institute for Health and Care Excellence United Kingdom guidelines in 2014 [38]. Relatedly, evidence suggests that the predictive ability of the HAS-BLED score outperformed that of other hemorrhage risk scores [42], which may have also solidified this score’s position in multiple society guidelines.

#### Updates to Widely Used Score Calculators

A fourth factor associated with high calculator popularity may be the use of calculators for clinical scores that constitute an update to an already existing high-profile clinical score. In our study, this is best exemplified by the CHA_2_DS_2_-VASc score, which was responsible for nearly one-third of stroke-related calculator page views between 2016 and 2018. This score was originally developed as a risk stroke prediction tool in atrial fibrillation that was improved compared with the existing CHADS_2_ score by incorporating several additional thromboembolic risk factors [17]. Dating back to the original score’s publication in Journal of the American Medical Association in 2001, practicing clinicians may have already been familiar with the concept of data-driven stroke risk prediction in atrial fibrillation by the time of the second score’s publication in 2009. This familiarity, in turn, may have cemented widespread acceptance of the CHA_2_DS_2_-VASc score’s viability as a clinical risk predictor.

#### Broad Applicability to Nonstroke Conditions

Applicability of calculators to multiple disease states may be additionally responsible for widespread use. For instance, we found that the second-most used cerebrovascular calculator was the MAP, which rose in relative page views between the 5-year period ending on December 31, 2015 and the end of the 3-year study period. Although MAP is often used to guide management of aneurysmal subarachnoid hemorrhage [43], our findings are likely attributable to the usefulness of MAP in diagnosing and managing several nonstroke states, such as sepsis, septic shock [44], and neurotrauma [45]. Indeed, in addition to subarachnoid hemorrhage, MDCalc metadata tags for the MAP calculator include both “sepsis” and “trauma.” Considering that severe sepsis and septic shock have higher yearly incidence than subarachnoid hemorrhage [46,47], the usefulness of MAP in the management of sepsis, rather than subarachnoid hemorrhage, may have been a more likely explanation for the high use of the MAP calculator during the study period. MAP is also less commonly used than systolic and diastolic blood pressure to guide the management of acute ischemic stroke [48,49] and intracerebral hemorrhage [50], thereby further supporting the theory that noncerebrovascular use cases were likely to be the primary drivers of high MAP calculator page views.

#### Score Use in High-Profile Randomized Trials

Inclusion of scores in high-profile randomized trials may also translate to high use of calculators associated with these scores. While the NIHSS score is not the sole factor in selecting patients for tissue plasminogen activator in acute ischemic stroke [49], the NIHSS was included in the first randomized controlled trial of tissue plasminogen activator for acute ischemic stroke [51] and incorporated as an inclusion criterion for several large randomized controlled trials demonstrating the effectiveness of mechanical thrombectomy for acute ischemic stroke due to anterior circulation large-vessel arterial occlusion [52-55], along with several confirmatory meta-analyses in 2015 and 2016 [56,57].

Factors other than guideline adoptions and validations for study publications may also explain the patterns we observed in our study, such as increased global use of medical calculators and increased popularity of the MDCalc service across all calculators. These factors remain difficult to measure. In addition, several health care institutions across the world already use internal calculator repositories for clinical care, which are variably integrated into institutional EHRs. While the worldwide extent of this practice remains poorly characterized, increasing prevalence of such repositories in the future is likely to reduce clinician reliance on and use of external calculators.

#### Duration of Calculator Availability

Calculators that are released earlier may also be more widely employed than more recently released calculators due to increased awareness or ongoing search engine optimization. In this study, incorporation into society guidelines may be the main factor explaining why CHA_2_DS_2_-VASc and HAS-BLED calculators were released the latest, yet demonstrated higher use than calculators that were released earlier, such as the MAP, CHADS_2_ and NIHSS. However, the unmistakable presence of calculators such as the MAP and NIHSS among the 5 most highly used stroke calculators may be a result of their earlier release dates.

#### Accessibility via Web Browser

Finally, our findings in stroke calculators suggest that web-accessible calculators may be more widely used than those that are primarily mobile app–based. These results are interesting, given that smartphone ownership in the United States has significantly increased since the early 2010s [58] and smartphone-based and tablet-based calculators are uniquely well suited to clinicians’ flexible and dynamic workflow requirements. However, MDCalc’s introduction of mobile apps in March 2016 (iOS) [59] and April 2017 (Android) should also be considered when interpreting our results [60]. Moreover, a significant proportion of the predominant web access we observed in our results may have occurred through mobile web browsers, which are highly prevalent in mobile devices and function identically to those found in stationary (eg, laptop or desktop) computers. However, because this study could not differentiate these different types of web access or the context in which these calculators were used, our findings cannot allow us to make definitive conclusions regarding the optimal mode or setting for stroke calculator deployment.

### Limitations

This study was limited by several factors. First, we restricted our analysis to calculators from a single platform. Because many other web-based CDS platforms are available for use, our results may not generalize to other platforms or to the entire community of medical professionals that actively use the 5 identified stroke-related scores in day-to-day practice. Second, because we used deidentified page view data for the study, we lacked user information that could permit a more detailed understanding of calculator use, such as discipline, medical speciality, level of training, as well as EHR, care setting, and disease states in which stroke-related calculators were used. For similar reasons, we have limited insight into whether MDCalc calculator use was potentially affected by alternative calculators embedded in care providers’ EHRs. Third, we did not investigate the effects these calculators, as CDS tools, had on aspects of clinician decision-making, such as diagnostic speed and accuracy, as studied by Abedin and colleagues [61]. We also did not investigate the relationship between calculator use and adherence to best practices or meaningful clinical outcomes. Finally, our study period was restricted to 3 years, which may have provided limited insights on use patterns and impacts on clinical care, especially as smartphone and mobile app usage have only become more ubiquitous since 2018.

### Conclusions

In this retrospective analysis, we demonstrated that the most commonly used stroke calculators were related to secondary stroke prevention in atrial fibrillation, blood pressure measurement, and computation of the NIHSS score. As medical calculators become increasingly important CDS tools, further studies should seek to understand optimal implementation and integration of these calculators into EHR systems and clinical care pathways. This can be achieved by incorporating a broader spectrum of calculator platforms, including platforms for user specialty and training and analyses of the behavior of clinicians during calculator use at the point of care. Additionally, considering our findings that stroke calculators were predominantly adopted in English-speaking countries and highly populated areas, further studies should aim to investigate barriers to adoption and whether translation of calculators into non-English languages may potentially improve calculator adoption.

## Appendix

Multimedia Appendix 1 Example of an MDCalc medical calculator webpage (CHA<sub>2</sub>DS<sub>2</sub>-VASc Score for Atrial Fibrillation Stroke Risk).

## Abbreviations

CDS: clinical decision support.
CHADS_2_: congestive heart failure, hypertension, 75 years of age or older, diabetes mellitus, and previous stroke or transient ischemic attack.
CHA_2_DS_2_-VASc: congestive heart failure, hypertension, 75 years of age and older, diabetes mellitus, previous stroke or transient ischemic attack, vascular disease, 65 to 74 years of age, female.
EHR: electronic health record.
HAS-BLED: hypertension, abnormal renal/liver function, stroke, bleeding history or predisposition, labile international normalized ratio, elderly, drugs/alcohol concomitantly.
MAP: mean arterial pressure.
NIHSS: National Institutes of Health Stroke Scale.
