# Supplementary figures and images for: Usage Patterns of Web-Based Stroke Calculators in Clinical Decision Support: Retrospective Analysis
Source: JMIR Med Inform. 2021 Aug 2;9(8):e28266. doi: 10.2196/28266 (PMC8369374; doi:10.2196/28266)

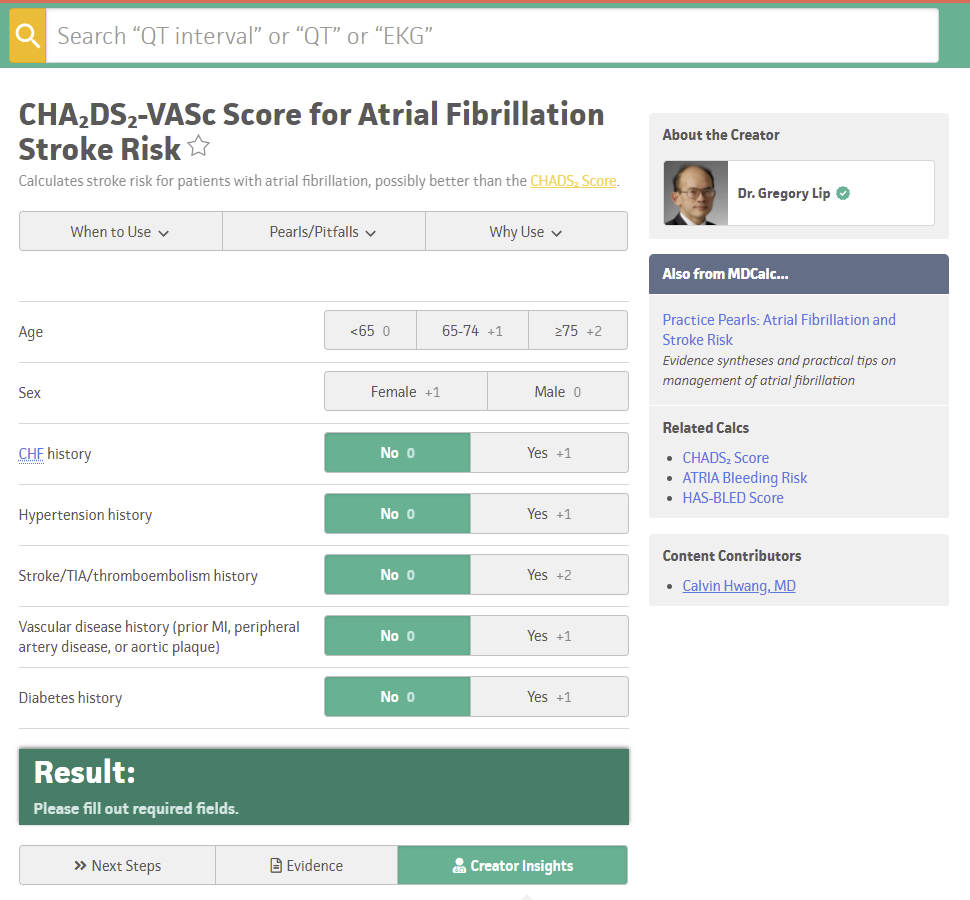

Supplement: Multimedia Appendix 1 [file medinform_v9i8e28266_app1.png]
